# Nine-Year Nationwide Environmental Surveillance of Hepatitis E Virus in Urban Wastewaters in Italy (2011–2019)

**DOI:** 10.3390/ijerph17062059

**Published:** 2020-03-20

**Authors:** Marcello Iaconelli, Giusy Bonanno Ferraro, Pamela Mancini, Elisabetta Suffredini, Carolina Veneri, Anna Rita Ciccaglione, Roberto Bruni, Simonetta Della Libera, Francesco Bignami, Massimo Brambilla, Dario De Medici, David Brandtner, Pietro Schembri, Stefania D’Amato, Giuseppina La Rosa

**Affiliations:** 1 Department of Environment and Health - Istituto Superiore di Sanità, 00161 Rome, Italy; marcello.iaconelli@iss.it (M.I.); giusy.bonannoferraro@iss.it (G.B.F.); pamela.mancini@iss.it (P.M.); carolina.veneri@guest.iss.it (C.V.); simonetta.dellalibera@iss.it (S.D.L.); bigfra91@gmail.com (F.B.); 2 Department of Food Safety, Nutrition and Veterinary Public Health, Istituto Superiore di Sanità, 00161 Rome, Italy; elisabetta.suffredini@iss.it (E.S.); dario.demedici@iss.it (D.D.M.); 3 Department Infectious Diseases, Istituto Superiore di Sanità, 00161 Rome, Italy; annarita.ciccaglione@iss.it (A.R.C.); roberto.bruni@iss.it (R.B.); 4 Consiglio per la ricerca in agricoltura e l’analisi dell’economia agraria (CREA), Research Centre for Engineering and Agri Food Processing, 24047 Treviglio, BG, Italy; massimo.brambilla@crea.gov.it; 5 Indipendent Researcher, 00156 Rome, Italy; restless.v2@gmail.com; 6 Regional Department for Health Activities and Epidemiological Observatory of the Sicilian Region, 90146 Palermo, Italy; p.schembri@regione.sicilia.it; 7 Ministry of Health, Directorate-General for Prevention, 00144 Rome, Italy; stefania.damato@sanita.it

**Keywords:** hepatitis E virus, sewage, sequencing, typing, PCR, prevalence

## Abstract

Hepatitis E virus (HEV) is an emerging causative agent of acute hepatitis worldwide. To provide insights into the epidemiology of HEV in Italy, a large-scale investigation was conducted into urban sewage over nine years (2011–2019), collecting 1374 sewage samples from 48 wastewater treatment plants located in all the 20 regions of Italy. Broadly reactive primers targeting the ORF1 and ORF2 regions were used for the detection and typing of HEV, followed by Sanger and next generation sequencing (NGS). Real-time RT-qPCR was also used to attempt quantification of positive samples. HEV RNA detection occurred in 74 urban sewage samples (5.4%), with a statistically significant higher frequency (7.1%) in central Italy. Fifty-six samples were characterized as G3 strains and 18 as G1. While the detection of G3 strains occurred in all the surveillance period, G1 strains were mainly detected in 2011–2012, and never in 2017–2019. Typing was achieved in 2 samples (3f subtype). Viral concentrations in quantifiable samples ranged from 1.2 × 10^3^ g.c./L to 2.8 × 10^4^ g.c./L. Our results suggest the considerable circulation of the virus in the Italian population, despite a relatively small number of notified cases, a higher occurrence in central Italy, and a noteworthy predominance of G3 strains.

## 1. Introduction

Hepatitis E virus (HEV) is one of the major causes of acute hepatitis in the world, commonly transmitted by the fecal–oral route through contaminated water and food. The World Health Organization (WHO) estimates that every year 20 million hepatitis E infections occur, resulting in over 3 million symptomatic cases (http://www.who.int/mediacentre/factsheets/fs280/en/). 

HEV infection develops in most individuals as self-limiting acute hepatitis, with mortality rates of around 1%. However, some individuals may develop fulminant hepatic failure, a severe condition that is frequently fatal. This complication is particularly common when the infection occurs in pregnant women, where mortality rates rise to up to 25% [1]. Furthermore, immunocompromised patients may progress to chronic hepatitis following an HEV infection [2].

Large-scale outbreaks of acute hepatitis occur in regions where HEV is endemic, such as Africa, Asia, and the Middle East. In such countries, HEV is primarily a waterborne illness associated with large epidemics due to contaminated water and water supplies and poor sanitation conditions [1,3]. In contrast, hepatitis E occurs as sporadic cases in Europe, Japan, and the USA, where HEV is spread mainly by zoonotic transmission, and domestic pigs and wild boars are considered main reservoirs [4,5]. Foodborne HEV is most commonly transmitted to humans consuming raw or undercooked pork products [6]. However, virus transmission can also follow the consumption of the meat of animals such as wild boar, deer, rabbits, and camels [7,8] as well as professional exposure to pigs and pork [9]. In low endemicity countries, hepatitis E was considered initially as a travel-associated disease, but in the last few years, an increasing number of autochthonous cases have been reported [8]. In Europe, the incidence of confirmed cases has steadily increased over the last decade [6]. The recognition of a high anti-HEV seroprevalence in a significant proportion of healthy individuals from nonendemic countries suggests widespread infection, which most likely occurs at a subclinical or asymptomatic level [10,11].

HEV is a member of the family *Hepeviridae*, genus *Orthohepevirus*, which is classified into four species: *Orthohepevirus* A–D [12,13]. All the human HEV genotypes (G1 to G4) belong to *Orthohepevirus* A, along with other genotypes (G5 and G6) that were isolated from wild boar [12], and G7 HEV isolated from camelids and a patient with chronic hepatitis [14]. Genotypes 1 (G1) and 2 (G2) are mainly restricted to humans, while genotypes 3 (G3) and 4 (G4) are isolated in both humans and animals (pig, deer, wild boar, rabbit) in developing and industrialized countries. Genotypes 1 (detected in Asia and Africa), and 2 (detected in Mexico and Africa), include strains from areas where the virus is endemic. G3 strain isolation occurs mostly in North America, Europe, South America, and Japan [15]. Genotype 4 has mainly been identified in China, Taiwan, Japan, and Vietnam, but a few cases have also been reported in Europe, including Italy [16,17,18].

The purpose of this study was to assess the occurrence and diversity of HEV in the Italian population through molecular analysis of urban sewage samples collected at wastewater treatment plants (WTPs). Environmental surveillance through the monitoring of sewer systems has been demonstrated as a useful auxiliary tool in the study of the epidemiology of viral pathogens, and an essential component of early warning systems. Indeed, a number of studies on the occurrence of HEV in urban sewages have been conducted in European countries, like Norway, Portugal, France, Spain, Sweden, Switzerland, UK, Greece, and Italy [19,20,21,22,23,24,25,26,27,28]. In the present study, however, a significant effort to provide a robust estimate of HEV circulation at a country level was undertaken through the application of a large-scale sampling scheme (approximately 1400 samples), over an extended temporal (nine years, from 2011 to 2019), and spatial scale (48 wastewater treatment plants distributed over the 20 regions of the country).

This allowed us to: 1) monitor HEV trends over time, both in terms of virus occurrence, and of circulating genotypes, comparing these trends with the data reported for notified human cases; and 2) assess the differences in the geographical occurrence of HEV, and identify hotspots of virus excretion, correlating them with data from the clinical setting. 

## 2. Materials and Methods

### 2.1. Sampling

**Figure 1:**
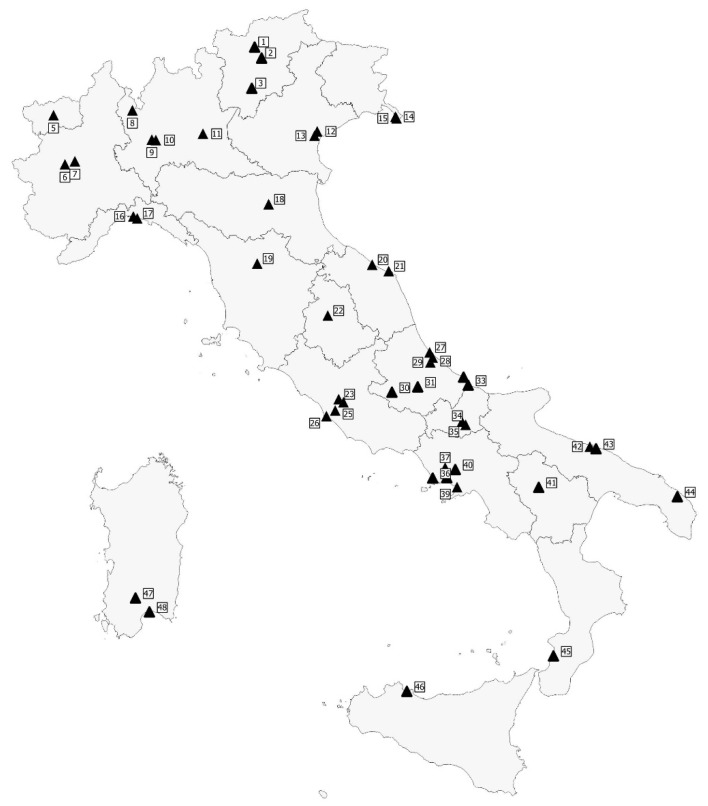
Geographic information system (GIS) map of the WTPs included in the study.

In the period 2011 to 2019, a total of 1374 raw sewage samples were collected from 48 WTPs distributed over the entire country and covering all the 20 Italian administrative regions: 529 samples were taken from 8 Regions of Northern Italy (Emilia Romagna, Friuli Venezia Giulia, Liguria, Lombardy, Piedmont, Trentino-South Tyrol, Veneto, and Aosta Valley), 590 from 5 Regions of Central Italy (Abruzzo, Latium, Marche, Tuscany, and Umbria) and 255 from 7 Regions of Southern Italy (Apulia, Basilicata, Calabria, Campania, Molise, Sardinia, and Sicily). Figure 1 shows a geographic information system (GIS) map of the WTPs included in the study. 

The environmental surveillance for HEV started within the framework of the 2011–2016 European Project *PREDEMICS* (Preparedness, prediction, and prevention of emerging zoonotic viruses with pandemic potential using multidisciplinary approaches - https://predemics.biomedtrain.eu/cms/). After that, the surveillance continued with the 2017–2019 national project “Hepatitis E, an emerging problem in food safety: ‘One Health’ approach for risk assessment” (CCM 2016 program) financed by the Italian Ministry of Health. 

All the samples, were registered in the PostgreSQL database (available to recorded users at https://cosmos.bio.uniroma1.it) and received a univocal ID code. Before concentration, samples underwent spiking with a known quantity of murine norovirus (MNV-1), used as a control process. 

### 2.2. Concentration and Treatment

Two different sewage treatment protocols were used during the 9-year surveillance; wastewater samples collected in 2011–2016 were analyzed, as previously described [29]. An aliquot of 20 mL of untreated wastewater (collected at the inlet collector, before treatment) were treated with 2 mL of 2.5 M glycine (pH 9.5), and incubated on ice for 30 min. The solution was then treated with 2.2 mL chloroform and centrifuged at 2300 g for 10 min, and the supernatant was recovered and measured. From 2017 onwards, with the start of the new project “Hepatitis E, an emerging problem in food safety: ‘One Health’ approach for risk assessment,” virus concentration was performed using the two-phase separation method (polyethylene glycol, (PEG)–dextran) according to the WHO guidelines for poliovirus environmental surveillance [30]. A sample of 250 mL of sewage underwent centrifugation at 4400 *g* for 30 min at 4 °C, and the resulting pellet was stored at 4 °C for the subsequent steps. The recovered supernatant, instead, was treated with 17 mL of 5 M sodium chloride, 142 mL of 29% polyethylene glycol 6000, and 20 mL of 22% dextran, and the mixture was transferred into a separation funnel and left overnight at 4 °C. After phase separation, about 10 mL of the lower phase, which included the interphase, was collected drop by drop into a sterile 50 mL centrifuge tube and was mixed with the previously saved pellet. Then, a 20% volume of chloroform was added to the recovered volume, thoroughly mixed by vortexing, and then centrifuged at 1000 *g* for 10 min at 4 °C. The supernatant was then recovered, and sample volumes recorded.

Viral nucleic acid extraction was carried out on 5 mL of chloroform-treated samples using the NucliSENS MiniMag (bioMerieux, Marcy l’Etoile, France) semi-automated extraction system, in accordance with the manufacturer’s instructions. Eluted RNA (100 μL) was stored in aliquots at −80 °C until molecular analysis. 

### 2.3. Reverse Transcription Polymerase Chain Reaction Nested Amplification (Nested RT-PCR)

A nested RT-PCR assay, using universal primers targeting the methyltransferase (MTase) gene of the ORF1 region [31], was used. This assay, that amplifies a 172 bp region of all HEV genotypes, was proven to be the most sensitive in a previous study comparing five different protocols [32].

PCR amplification was performed in a T100 thermal cycler (BioRad, Hercules, CA, USA) using MyTaq one-step RT-PCR kit (Bioline, Memphis, TN, USA). PCR reactions were prepared in a 25 µL mixture containing 12.5 µL of one-step mix, 1 µL (10 pmol) of each primer, and 2 µL of the extracted RNA. PCR cycling conditions were: reverse transcription at 45 °C for 20 min, 95 °C for 1 min, followed by 40 cycles of 95 °C for 10 s, 51 °C for 10 s and 72 °C for 30 s, and a final step at 72 °C for 5 min. After the first round of PCR amplification, one µL of PCR product underwent the nested amplification (35 cycles), performed using MyTaq red mix kit (Bioline). The PCR cycle included 1 min of initial denaturation at 95 °C; followed by 35 cycles of 95 °C for 15 s, 48 °C for 15 s, 72 °C for 10 s with a final elongation step of 5 min at 72 °C. The PCR products were visualized on 1.5% agarose gel stained with GelRed^®^ (Biotium, Fremont, CA, USA). Standard precautions were taken to prevent false-positive results.

All positive samples were reanalyzed using an assay targeting the ORF2 region [33,34,35], to allow a characterization of the strain, not only at the genotype level but also at the subtype level. 

### 2.4. Sequencing and Molecular Characterization 

PCR amplicons were purified using a Montage PCRm96 microwell filter plate (Millipore, Billerica, MA, USA) and were subjected to automated sequencing by BioFab Research (Rome, Italy), using the PCR amplification primers. As no ORF2 sequences could be obtained from the samples collected in 2017–2019 by Sanger sequencing, due to undetectable or unspecific PCR products, Next- Generation Sequencing (NGS) was also performed to sequence even low amounts of amplified specific fragments. All reactions of the ORF2 nested amplifications were pooled and sequenced by ultra-deep sequencing onto a MiSeq platform (Illumina, San Diego, CA, USA) as previously described [36]. The consensus sequence was constructed using Bowtie2 (Galaxy version 2.3.4.3+galaxy0, free software) as reads aligner and SSAKE (Galaxy version 0.010, MIT, Cambridge, USA) as contig assembler [37,38,39]. 

Phylogenetic relationships were studied by a neighbor-joining phylogenetic tree calculated on the maximum composite likelihood distances. Tree reliability was assessed through bootstrap analysis (1000 replicates) and considered significant linkage values >70%. The tree included the representative sequences belonging to genotypes 1 and 3 obtained from human and animal strains. All entries were reported with accession number, followed by host suffix (Sw, swine; Hu, human; WTP, wastewater treatment plant). Still unpublished sequences from clinical cases provided by the Italian National Reference Laboratory (NRL) for human Viral Hepatitis were reported as sample ID, followed by the year of isolation and administrative region origin.

Sequences were submitted to NCBI GenBank with the accession numbers LN624460 to LN624484, LN906799 to LN906809, and MN251630 to MN251657.

### 2.5. Real-Time RT-qPCR 

Real-time RT-qPCR analysis was conducted as previously described [40] on samples that were positive according to nested RT-PCR. Amplifications were carried out with the RNA UltraSense one-step qRT-PCR system (LifeTechnologies, Carlsbad, CA, USA), using 1.25 μL of UltraSense enzyme mix and the following concentrations for primers and probe: 500 nM for forward primer JVHEVF (5′-GGTGGTTTCTGGGGTGAC-3′), 900 nM for reverse primer JVHEVR (5′-AGGGGTTGGTTGGATGAA-3′), and 250 nM for probe JVHEVP-MGB (5′-FAM-TGATTCTCAGCCCTTCGC-MGB-3′) [41,42]. The thermal profile was as follows: reverse transcription at 50 °C for 60 min, inactivation at 95 °C for 5 min, followed by 45 cycles of 15 s at 95 °C, 1 min at 60 °C, and 1 min at 65 °C. Analyses were performed in duplicate reactions (5 μL of RNA each) on a Quant Studio 12K flex (ThermoFisher Scientific, Waltham, MA, USA), and the average concentration of the two replicate reactions was used for quantification. Two negative PCR controls were included in each run. PCR inhibition was ruled out using an external amplification control (in vitro synthesized RNA) and amplifications were considered acceptable if inhibition was ≤50%. A linearized plasmid containing the target sequence was used to generate the standard curve (dynamic range 1 × 10^0^ – 1 × 10^4^ copies/μL); curves with a slope lying between −3.1 and −3.6, and a R^2^ ≥ 0.98 were used for quantification. Quantitative values in raw sewage (expressed in genome copies, g.c./L) were calculated based on the fraction of the sample subjected to RNA extraction compared to its total volume after sample concentration.

### 2.6. Statistical Analysis

Statistical analysis (chi-square test) was performed using the Minitab 17 statistical software version 2010 (Minitab Ltd., Coventry, UK).

## 3. Results 

### 3.1. HEV Detection in Geographic Areas and Over Time

Results of HEV detection by nested PCR are reported in Table 1. Positive and negative controls, used to avoid the risk of false-positive or false-negative results, always gave the expected results. Overall, 74/1374 (5.4%, CI 95%; 4.3–6.7%) samples tested positive using the ORF1 assay: 23/529 (4.3%, CI 95%; 2.9–6.5%) in Northern Italy, 42/590 (7.1%, CI 95%; 5.3–9.5%) in Central Italy, and 9/255 (3.5%, CIm95%; 1.8–6.7%) in Southern Italy. A statistically significant difference (chi-square *p* value = 0.042) was present in HEV detection in the three geographic areas. No positive samples were detected in 7 regions (Basilicata, Campania, Liguria, Sardinia Tuscany, Umbria, and Valle d’Aosta), while in the remaining 13 regions HEV occurrence ranged from 3.0% (Veneto) to 14.2% (Molise). 

**Figure 2:**
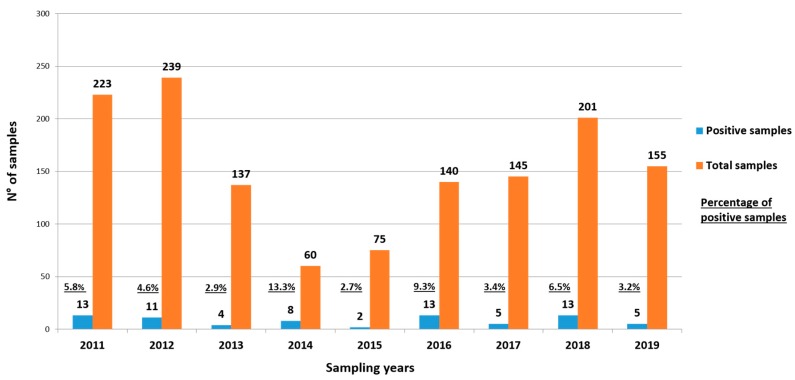
The distribution of positive samples (blue columns) vs total samples (orange columns) over the sampling period (2011–2019). The percentage of positive samples for each year is indicated above the absolute number.

Positive samples were found in all the years of the monitoring, ranging from 2.7% to 13.3% (Figure 2). 

### 3.2. HEV Quantification in Positive Sample 

Seventy-two samples underwent analysis by real-time RT-qPCR (Table 2). Thirty-two samples were below the detection limit, and most of the positive samples (31 out of 40) were below the analytical LOQ of 1.1 × 10^3^ genome copies (g.c.)/L, with estimated concentrations ranging from ~20 to 9.9 × 10^2^ g.c./L. Quantifiable samples ranged from 1.2 × 10^3^ g.c./L to 2.8 × 10^4^ g.c./L, with a median value of 3.2 × 10^3^ g.c./L. 

### 3.3. HEV Genotyping

**Figure 3:**
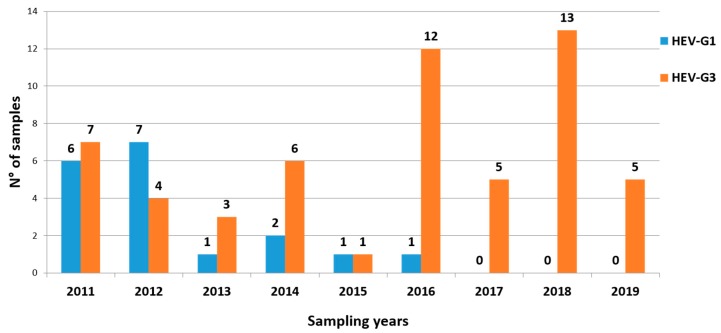
The number of HEV genotype 1 (blue columns) and genotype 3 (orange columns) positive samples detected during the study period (2011–2019).

Of the 74 positive samples, 56 were characterized as G3 and 18 as G1. While G3 sequences were detected in all the 9 years of monitoring (Figure 3), G1 sequences were mainly detected in 2011–2012 (13 of 18; 72%). Thereafter, only five G1 samples were found between 2013 and 2016, while in the triennium 2017–2019 only G3 sequences were detected.

**Figure 4:**
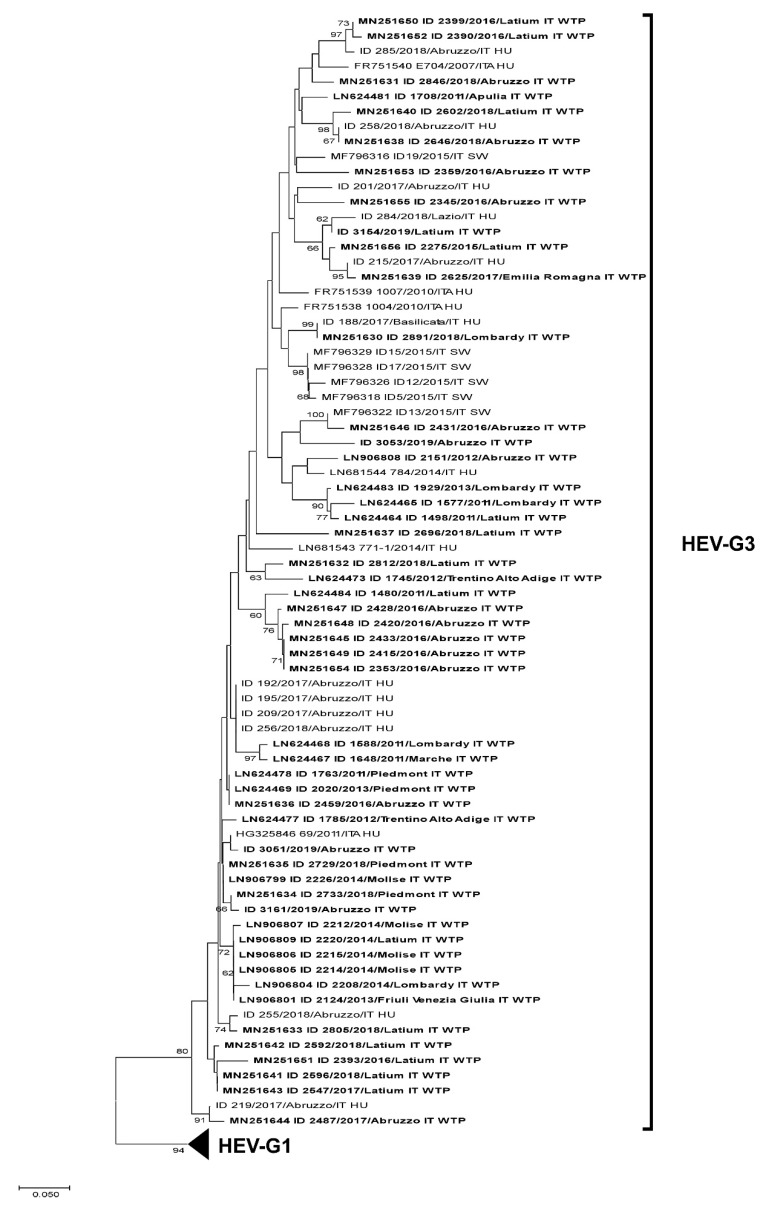

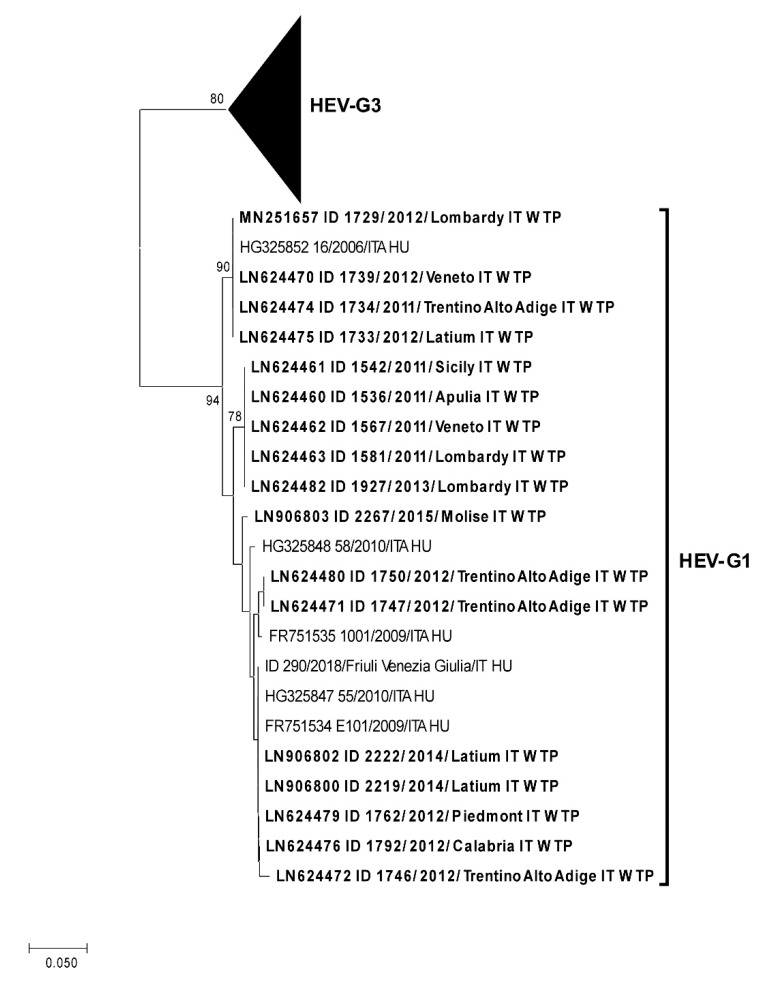
(**top**) Neighbor-joining phylogenetic tree of HEV-G3 sequences. (**bottom**) Neighbor-joining phylogenetic tree of HEV-G1 sequences.

A phylogenetic tree of the Mtase-ORF1 fragment obtained from the HEV strains detected in this study is shown in Figure 4A,B. The tree includes, in addition to the study sequences, clinical, environmental, and animal (swine) sequences previously detected in Italy. According to the sequencing results, the phylogenetic analyses showed that the strains were grouped into two main clusters, related to HEV G1 and G3. HEV G3 strains showed high sequence identity with human sequences detected in autochthonous cases (no travel history) and swine in Italy, and most of the G1 strains showed 100% nt identity with Italian strains detected in patients traveling in endemic areas.

An attempt to further characterize the positive samples was performed using broad-range primers targeting the ORF2 gene, but only 2 of these samples provided amplification in this region (ID sample 2222 and 2275, both collected from WTPs in Rome, in 2014 and 2015, respectively), and were characterized as subtype 3f by Sanger sequencing. NGS performed on pooled PCR amplicons collected in years 2017–2019 detected another G3f sequence. 

## 4. Discussion 

In Italy, the circulation of HEV has been demonstrated since the 1980s. Sporadic cases have been reported [18,32,35,43], related both to traveling to endemic countries and autochthonous. The latter cases were associated with consumption of game meat, dried pork liver sausage, and raw-dried wild boar sausages [44]. 

As for animal HEV, it has been demonstrated that HEV circulates largely within pig herds in Italy [45,46,47,48,49]. Genotype 3 is widespread among Italian pigs, even though genotype 4 was also reported in swine farms in Northern Italy [17]. HEV RNA has also been detected in pig slurry collected in Italian farms, showing a high degree of identity with strains detected in autochthonous HEV cases [50]. Moreover, different studies have demonstrated the presence of HEV RNA in liver samples from the wild boar population [40,51,52,53] and even in sheep serum and fecal samples [54]. 

The present study aimed to provide information on the occurrence and diversity of HEV in the Italian population through molecular analysis of urban sewage samples collected at WTPs. A number of studies have investigated HEV in urban sewages in nonendemic countries [19,23,26,28,55,56,57,58,59,60,61,62,63,64]. However, most of these studies are limited in time and conducted on a small number of samples. The present study, instead, describes a large-scale investigation, with a significant number of samples, a nationwide geographic coverage, and a nine-year observation period. 

HEV RNA detection in sewage occurred throughout the years 2011–2019 (mean detection frequency 5.3%) with higher occurrence in 2014 (13.3%). In a study conducted following an HEV outbreak on a small French island, Miura et al. estimated that if 1–4% of inhabitants connected to a WTP were infected with HEV, the virus would be detectable in urban wastewater [57]. While it is not possible to directly infer these conclusions to WTPs of different capacities and size, our results on the occurrence of HEV in sewage suggests the considerable circulation of the virus in the Italian population, despite the relatively small number of notified cases. During the period from 2011 to the first semester of 2019 only 323 cases of acute viral hepatitis were reported in the Italian integrated epidemiological system for acute viral hepatitis (SEIEVA) managed by the Italian Institute of Health (ISS) (https://www.epicentro.iss.it/epatite/bollettino/Bollettino-4-marzo-2019.pdf and https://www.epicentro.iss.it/epatite/bollettino/Bollettino-n-5-novembre-2019.pdf), with a trend of autochthonous cases on the rise over the study period (7 in 2011 vs 45 in 2018). The discordance between the relatively constant HEV detection in Italian sewages and the low incidence of HEV infection in humans may be associated with nondetection of mild, subclinical, or asymptomatic cases, which, however, shed virus into local sewage systems and contributed to virus circulation. This phenomenon is recognized as the “surveillance pyramid” [65]; because of under and misdiagnoses, clinical surveillance is likely to only capture the tip of the iceberg of viral diseases, generally corresponding to the hospitalized or laboratory diagnosed cases, while the full extent of disease can only be captured through studies addressing incidence and etiology at a community level. In this sense, the monitoring of urban wastewaters provides a powerful tool for indirect assessment of the community health status.

Comparing to data available from other European countries, the percentages obtained in the present study are similar to those found in Norway and Portugal (8.0% and 6.7%, respectively) [19,20], but lower than those documented in France (22–25%) [21], Spain (13.5–43.5%) [22,23,24], Sweden (33.0%) [25], Switzerland (24.1–32.3%) [21], and UK (93.0%) [26]; on the other hand, no positive results were detected in samples from Greece [23,27]. All these studies, however, are difficult to compare because they were very different in terms of the period of sampling, sample size, sample volume and processing, and molecular methods used. In our study, during the 9-year surveillance period, a shift of the method for concentration of viruses took place, moving from the “glycine-chloroform” to the “two-phase separation” method recommended by WHO for poliovirus environmental surveillance, which is considered the gold standard for the concentration of viruses from sewage. Both methods were applied with the use of a control virus, that was added to the sample before virus concentration in order to monitor the efficiency of the entire procedure and ensure the quality of the analytical results. Indeed, no statistically significant difference was detected in the observed period based on the concentration method used (5.8% of positive samples with “glycine-chloroform” and 4.6% with the “two-phase separation”; *p* value = 0.33). 

As for quantitative data, we found overall low HEV concentrations in raw sewage (range of concentrations ~20 to 2.8 × 10^4^ g.c./L), with only nine samples exceeding 10^3^ g.c./L and a median value of quantifiable samples of 3.2 × 10^3^ g.c./L. Few studies have been performed on HEV quantitative levels in raw sewage, and comparison of available data is hindered by the diversity of analytical methods and by WTP capacity. In three studies carried out in Switzerland, Sweden, and Norway [19,21,25] most of the positive samples were below the corresponding LOQs and, overall, only two samples were quantified with concentrations of 7.8 × 10^4^ c.g./L [21] and 4.5 × 10^4^ c.g./L (25). Miura and colleagues [57] in their study performed during an HEV outbreak on an island, reported virus concentrations in inlet sewage up to 6.3 × 10^5^ c.g./L after the outbreak, with values decreasing to 10^3^ c.g./L within a month. Therefore, the quantitative data of the present study seem to confirm that, during the observation period, the detection of HEV occurred at concentrations lower than those presumably associated with relevant outbreaks in the populations.

Of all the positive samples detected, 75.6% were G3 and 24.3% G1. While G3 strains were consistently detected in the 9 years of surveillance, G1 were mainly detected in 2011 (6 samples) and 2012 (7 samples, outnumbering G3), and then sporadically, in 2013–2016. In the last three years, only G3 strains were detected. Genotype 1 is the most common cause of hepatitis E in regions with a lower socioeconomic status, where it is predominantly spread via contaminated potable water. In Italy, infections with HEV G1 have been documented in immigrants from countries at risk after returning from a stay in their country of origin, as well as in Italian people traveling from highly endemic countries [32,35,66]. The environmental data provided in this study reflect those from clinical surveillance. Indeed, of the total 285 cases documented in SEIEVA in 2011–2018 (https://www.epicentro.iss.it/epatite/bollettino/Bollettino-4-marzo-2019.pdf), 23.5% were imported (these are most probably caused by HEV G1), and 76.5% were autochthonous (caused by G3). The sporadic circulation of genotype 1, along with the predominance of genotype 3, is consistent with reports in the literature about other industrialized countries [22,23]. The predominance of G3 cases is likely to result in a higher concentration of G3 molecules in sewage samples compared to G1, leading to a predominance of G3 PCR amplification products and to the apparent absence of G1 in some years. Unfortunately, only two of the G3 positive samples could be typed by sequencing the ORF2 region. The possible explanations for the negative ORF2 detection in the vast majority of positive samples, besides the lower sensitivity of the ORF2 assay, include the possibility of RNA damage due to the long storage time before testing, despite the efforts for sample preservation. Moreover, being that the ORF2 assay is an amplification providing a longer template compared to ORF1 (>450 bps vs 172 bp), amplification from environmental samples, which often contain inactivated viruses and fragmented RNA, may be more difficult to achieve. The two positive samples were both characterized as G3f, which is also the predominant type reported in the same period in the clinical cases tested by the Italian NRL for human viral hepatitis (unpublished data). A G3f sequence was also detected by NGS in 2017–2019 pooled samples.

Geographically, HEV identification occurred in 13 Italian Regions out of 20. In the seven regions where the virus has not been identified, we cannot be sure whether such an outcome relates to the number of samples that could be collected. However, it should also be considered that the high variability of HEV occurrence in sewage, besides the kind of influent the WTP is fed with, strictly depends on the sampling volumes and the time of collection [67]. Indeed, due to the long surveillance period, few WTPs provided samples continuously over the nine years, while the vast majority provided samples in limited periods within this time, resulting in an unequal number of samples by WTP and by administrative region. Therefore, a detailed comparative analysis of the distribution of positive samples by WTP and administrative region was not feasible. Generally speaking, higher percentages of positive samples were detected in Central Italy (a difference found to be statistically significant) and, in particular, in Latium, Marche, and Abruzzo. These data were in line with clinical data; indeed, according to data from the Italian NRL for human viral hepatitis (ISS, Rome), 67% of the confirmed cases (IgM+ and/or HEV RNA +) of HEV in Italy are from Latium and Abruzzo (unpublished data). 

Serological data fosters the marked heterogeneity of seroprevalence in the different Italian geographic areas. As a matter of fact, rates in the general population range from 0.12% to 17.8% [68,69,70,71,72,73]. The different sensitivity of the anti-HEV detection systems used in different studies (the older ones using kits with lower sensitivity) partially accounts for the observed heterogeneity. However, recent testing on 10,011 blood donors throughout Italy, using the same highly sensitive detection kit, showed that a true geographical prevalence heterogeneity exists, with very high rates of infection in some areas of Central Italy [74]. Those data confirmed a previous study whose results pointed at Abruzzo as a hyper-endemic region, with a seroprevalence rate of 49% among blood donors [75]. Abruzzo and Latium can be therefore considered hot spot regions for HEV circulation. It may be relevant that these administrative regions have traditional dishes based on raw/undercooked pork products, in particular liver sausages that, both raw and dried, may contain HEV [76]. Recently, an outbreak occurred in the bordering region Marche, linked to the consumption of a locally produced raw pork liver sausage [77].

The occurrence of HEV in urban wastewater raises the question of whether sewage-contaminated surface waters can be responsible for HEV waterborne transmission. A recent review by Fenaux et al. [67] highlighted the need to investigate the possible transmission of the hepatitis E virus by water in high-income countries. Indeed, HEV has been detected in different water matrices even in these countries; moreover, HEV survival in water is similar to other enteric viruses since it seems that the virus has similar resistance to inactivating factors like heat, UV, and chlorine [67]. In Italy, HEV has been recently detected in river waters receiving wastewater discharges [78]. Moreover, HEV strains have been isolated from shellfish, marine waters, and underwater sewage discharges [79,80]. 

## 5. Conclusions

This surveillance of human wastewaters allowed us to generate data on the circulation of HEV in the general population in Italy. HEV RNA, genotype 1 and 3, were detected at the WTP entrance, with the highest rates reported from the central regions of Italy. Viral sequences were genetically close to those reported in clinical specimens. A marked predominance of G3 strains was detected as expected, however, G1 strains were also identified, except in years 2016–2019, pointing to the circulation of this genotype also in nonendemic areas.

## Figures and Tables

**Table 1:** Nested RT-PCR results according to Region and geographic area.

Area	Region	Number of Tested Samples	Number of Positive Samples (%)
**North**	Emilia Romagna	35	2 (5.7)
	Friuli Venezia Giulia	11	1 (9.1)
	Liguria	25	0 (0)
	Lombardy	162	8 (4.9)
	Piedmont	82	5 (6.1)
	Trentino-South Tyrol	144	5 (3.5)
	Veneto	66	2 (3.0)
	Aosta Valley	4	0 (0)
***subtotal***		**529**	**23 (4.3)**
**Center**	Tuscany	24	0 (0)
	Umbria	5	0 (0)
	Latium	270	20 (7.4)
	Marche	14	1 (7.1)
	Abruzzo ^a^	277	21 (7.2)
***subtotal***		**590**	**42 (7.1)**
**South**	Apulia	40	2 (5.0)
	Basilicata	12	0 (0)
	Calabria	25	1 (4.0)
	Campania	89	0 (0)
	Molise	35	5 (14.2)
	Sardinia	24	0 (0)
	Sicily	30	1 (3.3)
***subtotal***		**255**	**9 (3.5)**
**Total**		**1374**	**74 (5.4)**

^a^ Abruzzo is officially a region of Southern Italy, because of its historic association with the Kingdom of the Two Sicilies, but is geographically located in Central Italy, bordering Lazio and Marche administrative regions.

**Table 2:** Genotyping and HEV concentration in positive samples.

Sample ID	City	Sampling Date (day/month/year)	Region	Area	Genotype	Concentration (g.c./L)
1480	Rome	18/05/2011	Latium	Center	G3	7.9 × 10^2^
1484	Rome	18/05/2011	Latium	Center	G3	nt
1498	Rome	20/06/2011	Latium	Center	G3	9.2 × 10^2^
1536	Bari	01/08/2011	Apulia	South	G1	<LOD
1542	Palermo	08/03/2011	Sicily	South	G1	<LOD
1567	Venice	27/09/2011	Veneto	North	G1	nt
1577	Milan	10/09/2011	Lombardy	North	G3	nt
1581	Milan	21/09/2011	Lombardy	North	G1	<LOD
1588	Milan	16/11/2011	Lombardy	North	G3	7.3 × 10^2^
1648	Ancona	18/11/2011	Marche	Center	G3	<LOD
1708	Bari	06/12/2011	Apulia	South	G3	5.9 × 10^2^
1729	Brescia	01/06/2012	Lombardy	North	G1	<LOD
1733	Rome	31/05/2012	Latium	Center	G1	9.9 × 10^2^
1734	Bologna	22/11/2011	Emilia Romagna	North	G1	**2.3 × 10^3^**
1739	Venice	23/04/2012	Veneto	North	G1	3.7 × 10^1^
1745	Trento	07/02/2012	Trentino Alto Adige	North	G3	1.3 × 10^2^
1746	Trento	07/03/2012	Trentino Alto Adige	North	G1	<LOD
1747	Trento	04/04/2012	Trentino Alto Adige	North	G1	2.0 × 10^2^
1750	Trento	18/04/2012	Trentino Alto Adige	North	G3	<LOD
1762	Turin	24/05/2012	Piedmont	North	G1	<LOD
1763	Turin	20/12/2011	Piedmont	North	G1	2.0 × 10^1^
1785	Bolzano	01/05/2012	Trentino Alto Adige	North	G3	<LOD
1792	Reggio Calabria	08/04/2012	Calabria	South	G1	nt
1927	Milan	14/03/2013	Lombardy	North	G1	**1.2 × 10^3^**
1929	Milan	26/06/2013	Lombardy	North	G3	5.5 × 10^1^
2020	Collegno	06/03/2013	Piedmont	North	G3	3.1 × 10^1^
2124	Trieste	02/10/2013	Friuli Venezia Giulia	North	G3	nt
2151	Pescara	03/04/2012	Abruzzo	Center	G3	1.9 × 10^2^
2208	Milan	28/08/2014	Lombardy	North	G3	2.9 × 10^1^
2212	Campobasso	18/03/2014	Molise	South	G3	<LOD
2214	Campobasso	10/02/2014	Molise	South	G3	<LOD
2215	Campobasso	06/08/2014	Molise	South	G3	**1.4 × 10^3^**
2219	Rome	16/07/2014	Latium	Center	G1	7.8 × 10^1^
2220	Rome	26/07/2014	Latium	Center	G3	<LOD
2222	Rome	04/08/2014	Latium	Center	G1	nt
2226	Campobasso	20/10/2014	Molise	South	G3	nt
2267	Campobasso	30/03/2015	Molise	South	G1	<LOD
2275	Rome	10/07/2015	Latium	Center	G3	<LOD
2320	San Salvo	27/01/2016	Abruzzo	Center	G1	9.0 × 10^1^
2345	Vasto	03/02/2016	Abruzzo	Center	G3	7.5 × 10^2^
2353	Pescara	15/03/2016	Abruzzo	Center	G3	3.4 × 10^2^
2359	Montesilvano	15/03/2016	Abruzzo	Center	G3	1.0 × 10^2^
2390	Rome	09/05/2016	Latium	Center	G3	4.4 × 10^2^
2393	Rome	30/05/2016	Latium	Center	G3	<LOD
2399	Rome	25/07/2016	Latium	Center	G3	2.7 × 10^1^
2415	Pescara	11/07/2016	Abruzzo	Center	G3	3.8 × 10^2^
2420	Pescara	26/09/2016	Abruzzo	Center	G3	2.8 × 10^2^
2428	Chieti	08/07/2016	Abruzzo	Center	G3	<LOD
2431	Chieti	26/08/2016	Abruzzo	Center	G3	<LOD
2433	Chieti	29/09/2016	Abruzzo	Center	G3	1.3 × 10^2^
2459	Pescara	12/12/2016	Abruzzo	Center	G3	<LOD
2487	Sulmona	26/06/2017	Abruzzo	Center	G3	nt
2492	Vasto	19/04/2017	Abruzzo	Center	G3	2.5 × 10^2^
2501	Rome	18/07/2017	Latium	Center	G3	8.0 × 10^1^
2547	Rome	22/12/2017	Latium	Center	G3	2.0 × 10^2^
2625	Bologna	08/08/2017	Emilia Romagna	North	G3	6.3 × 10^1^
2592	Rome	25/01/2018	Latium	Center	G3	<LOD
2596	Rome	22/02/2018	Latium	Center	G3	nt
2602	Rome	21/03/2018	Latium	Center	G3	<LOD
2646	Vasto	14/03/2018	Abruzzo	Center	G3	4.3 × 10^1^
2696	Rome	27/06/2018	Latium	Center	G3	2.0 × 10^2^
2729	Turin	26/04/2018	Piedmont	North	G3	6.4 × 10^2^
2733	Turin	13/06/2018	Piedmont	North	G3	<LOD
2805	Rome	17/04/2018	Latium	Center	G3	**3.2 × 10^3^**
2812	Rome	01/12/2018	Latium	Center	G3	<LOD
2842	Avezzano	04/07/2018	Abruzzo	Center	G3	<LOD
2846	San Salvo	09/07/2018	Abruzzo	Center	G3	**5.3 × 10^3^**
2852	Vasto	12/07/2018	Abruzzo	Center	G3	<LOD
2891	Milan	10/07/2018	Lombardy	North	G3	**2.8 × 10^3^**
3051	Vasto	17/04/2019	Abruzzo	Center	G3	**6.9 × 10^3^**
3053	Vasto	17/06/2019	Abruzzo	Center	G3	**2.8 × 10^4^**
3093	Pescara	15/05/2019	Abruzzo	Center	G3	**9.5 × 10^3^**
3154	Rome	01/05/2019	Latium	Center	G3	5.2 × 10^2^
3161	San Salvo	31/10/2019	Abruzzo	Center	G3	<LOD

nt **=** not tested due to insufficient RNA. Quantitative values in bold were above the analytical LOQ (1.1 × 10^3^ g.c./L); other values are provided as estimated concentrations.
